# A randomised double blind placebo-controlled clinical trial to evaluate the effects of Liv-Pro- a food supplement for Non-Alcoholic Fatty Liver Disease

**DOI:** 10.1016/j.jaim.2025.101196

**Published:** 2025-11-11

**Authors:** Anchala Ishani Kuruppu, Uditha Prabhath Liyanage, Aruni Chamika, Priyani Asoka Paranagama, Lankani Hettigoda

**Affiliations:** a Institute for Combinatorial Advanced Research and Education, General Sir John Kotelawala Defence University, Ratmalana, Sri Lanka; b Department of Statistics and Computer Science, University of Kelaniya, Sri Lanka; c Siddhalepa Ayurveda Hospital (Pvt) Ltd, Mount Lavinia, Sri Lanka; d Department of Chemistry, Faculty of Science, University of Kelaniya, Sri Lanka; e Hettigoda Industries (Pvt) Ltd, Ratmalana, Sri Lanka

**Keywords:** Non-alcoholic fatty liver disease (NAFLD), Fatty liver, Ayurveda, Traditional Sri Lankan medicine, Food and dietary supplements

## Abstract

**Background:**

Non-alcoholic Fatty Liver Disease (NAFLD) involves excessive fat build-up in the liver unrelated to alcohol use. This study aimed to evaluate the clinical efficacy and safety of Liv-Pro capsules, a formulation combining *Osbeckia octandra* and *Aloe vera*, in managing NAFLD. This is the first report of the clinical trial using this combination against NAFLD.

**Methods:**

A randomized, double-blind, placebo-controlled clinical trial was conducted with 30 NAFLD patients from February 1, 2021 to March 31, 2023. Participants received either Liv-Pro or a placebo (two capsules once a day) for two months. Medical assessments at baseline, one month, and two months included liver function tests (ALT and AST), abdominal ultrasound scan, serum creatinine analysis, and lipid profile assessment. All patients were advised on lifestyle modifications.

**Results:**

Liv-Pro significantly reduced ALT levels after one month (p = 0.049) and two months (p = 0.048) compared to baseline. AST levels also decreased significantly in the first (p = 0.047) and second months (p = 0.046), showing a consistent decline. Liv-Pro reduced total cholesterol, triglycerides, and Low-density lipoprotein (LDL) levels significantly after one and two months (p < 0.05) compared to baseline measurements. Abdominal ultrasound scans showed a significant decrease in hepatic fat content after two months (p = 0.020). Serum creatinine levels remained stable throughout the treatment period.

**Conclusion:**

Liv-Pro was well-tolerated with no reported side effects, suggesting its potential as a safe and effective therapeutic option for NAFLD. Continuous administration of Liv-Pro is recommended for sustained efficacy.

## 1 Introduction

Non-alcoholic fatty liver disease (NAFLD) is a silent and progressive disorder that has a surplus of fat in the liver that is not a result of excessive alcohol consumption [[1], [2]]. It is a common disorder around the globe with increased mortality [3]. NAFLD occurs in tandem with metabolic abnormalities, and it covers a wide range of ailments from fat deposition, along with mild or no inflammation which transfers to non-alcoholic steatohepatitis (NASH), to cirrhosis [4]. NAFLD is prevalent globally, with an estimated 38 % of adults and 10 % of children and adolescents affected between 2016 and 2019. This marks a significant increase from 25.3 % in 2006 [[5], [6], [7]]. The condition is more common in males (40 %) than females (26 %) [8]. The rising prevalence of NAFLD is linked to unhealthy diets and sedentary lifestyles associated with industrialization and urbanization [9].

NAFLD is also a significant concern in Asia, with a high prevalence in Sri Lanka, where 33 % of the adult urban population and 8.4 % of adolescents are affected [4]. NAFLD can lead to health complications such as cardiovascular disease, liver cancer, and type 2 diabetes. Further, Sri Lankan NAFLD patients have a nearly two-fold greater risk of developing type 2 diabetes. Furthermore, NAFLD is often associated with obesity, type 2 diabetes, hypertension, and dyslipidemia, all of which increase cardiovascular risk and comprise metabolic syndrome [10,11]. At present, NAFLD management primarily involves lifestyle changes such as diet, exercise, and increasing insulin sensitivity [12]. However, maintaining these changes is challenging, leading to low patient compliance [13,14].

Ayurvedic literature categorizes liver disorders under conditions such as ‘Pandu’ (anemia), ‘Kamala’ (jaundice), ‘Raktapitta’ (blood disorders), and ‘Udara Roga’ (abdominal diseases). In its early stages, the accumulation of ‘Medas’ (fat tissue) in the liver is recognized as a form of ‘Udara Roga’, caused by imbalances in the ‘Kaphaja’ (phlegm-related) and ‘Vataja’ (air-related) doshas, often initiated by ‘Mandagni’ (poor digestion) and improper dietary and lifestyle habits. Over time, these early pathological changes may progress into ‘Yakriddalyudara’ (liver-related abdominal diseases), which is characterized by excessive accumulation of ‘Medas’ in the liver. This stage closely aligns with the modern understanding of fatty liver disease, where fat deposits disrupt liver function. The excessive accumulation of ‘Medas’ in fatty liver disease results from the improper conversion of ‘Medo Dhatu’ (fat tissue) from ‘Mamsa Dhatu’ (muscle tissue) due to the dysfunction of ‘Medo Dhatu Agni’ (tissue-specific metabolism) [15]. This impaired metabolic process gives rise to ‘Udara Roga’, with ‘Mandagni’ playing a pivotal role in producing ‘Ama’ (toxins) that reduce the liver's ability to metabolize and detoxify substances. This cascade of events slows down metabolism, disrupts fat processing, and promotes fat deposition in the liver, ultimately leading to ‘Yakriddalyudara’ [16]. Ayurveda further explains that the excessive accumulation of ‘Medas’ obstructs the flow of vital nutrients (Rasa) and energy (Ojas), leading to diminished physical and mental functions. The improper conversion of ‘Medas’ into ‘Asthi Dhatu’ (bone tissue) is hindered due to ‘Medovaha Srotas Avarodha’ (blockage in fat transport channels) during ‘Dhatu Parinama’ (tissue transformation) [15]. This blockage results in ‘Medo Dhatu Vruddhi’ (excessive fat accumulation). The ‘Medovaha Srotas’, rooted in the ‘Vrukka’ (kidneys) and ‘Vapa Vaha’ (omentum), play a critical role in visceral fat accumulation. Obstruction in these channels leads to fat deposition around abdominal organs, further impairing the transformation of ‘Medo Dhatu’ into other tissues. This contributes not only to ‘Atisthaulya’ (obesity) but also to the pathogenesis of metabolic disorders, including fatty liver disease.

In the modern era, ‘Atisthaulya’ (obesity) has become a significant public health challenge. Ayurveda identifies ‘Atisthaulya’ as a pathological condition involving the excessive accumulation of ‘Medo Dhatu’ and ‘Mamsa Dhatu’ beyond normal limits. Classified as one of the ‘Ashta Nindita Purusha’ (eight undesirable conditions), ‘Atisthaulya’ is linked to numerous physical and psychological complications [15]. The harmful effects of excessive ‘Medas’ are encapsulated in the following Ayurvedic verse:

“Medasavrta-margatvat pusyantyanyena datavah

Medastu ciyate tasmat aśakthah sarva-karmasu”

This verse emphasizes that when ‘Medas’ obstructs bodily channels, the distribution of vital nutrients (Rasa) and energy (Ojas) becomes inadequate [17]. This results in fat accumulation, rendering a person incapable of performing physical and mental tasks efficiently.

The vitiation of ‘Medas’ adversely impacts other ‘Dhatus’, particularly ‘Rasa Dhatu’, which is the precursor of ‘Ojas’ [18]. Depletion of ‘Ojas’ weakens metabolic processes, exacerbating conditions like ‘Madhumeha’ (diabetes mellitus), where blood sugar regulation is impaired [19]. Furthermore, the obstruction of ‘Srotas’ (channels) by ‘Medas’ amplifies fat accumulation, aggravates ‘Kapha Dosha’, and contributes to the development of ‘Yakriddalyudara’ (fatty liver disease) and ‘Madhumeha’ (diabetes mellitus). This progression underscores the intricate relationship between impaired digestion, disturbed metabolism, and fat accumulation in the pathogenesis of fatty liver disease/NAFLD. By recognizing these interconnections, Ayurveda provides a holistic framework for understanding and addressing the metabolic imbalances underlying fatty liver disease/NAFLD, ‘Medo Vruddhi’, and ‘Madhumeha’.

Sri Lanka is home to around 3500 floral species, and of these, one quarter is endemic to the country. Most of these plants possess medicinal properties [20]. The island currently sustains a robust traditional medicine system, serving approximately 70 % of its population. This system encompasses Ayurveda, Siddha, Unani, and Deshiya Chikitsa (indigenous medical practices), where most systems utilize medicinal plants as the primary treatment source. The indigenous medical system, which has been practiced in Sri Lanka for thousands of years, predates the introduction of Ayurveda from India. Over time, both systems have been partially integrated in practice in certain treatment areas [21]. The objective of the present study was to test the safety and efficacy of the food supplement named ‘Liv-Pro’ developed with two medicinal plants, one of which is used only in the Sri Lankan iBackspaceIndigenous medical system to treat liver issues in the management of NAFLD. This study was conducted as a randomized, double-blind, placebo-controlled clinical trial involving patients diagnosed with non-alcoholic fatty liver disease (NAFLD). Diagnosis was based on elevated alanine aminotransferase (ALT)/Serum glutamate pyruvate transaminase (SGPT) and aspartate transaminase (AST)/Serum glutamic oxaloacetic transaminase (SGOT) levels, along with a confirmation by an abdominal ultrasound scan. Participants also exhibited lifestyle-related risk factors, including a high-fat diet, sedentary behavior, and/or increased body mass index (BMI).

## 2 Materials and methods

### 2.1 Identification of plant material

**Fig. 1:**
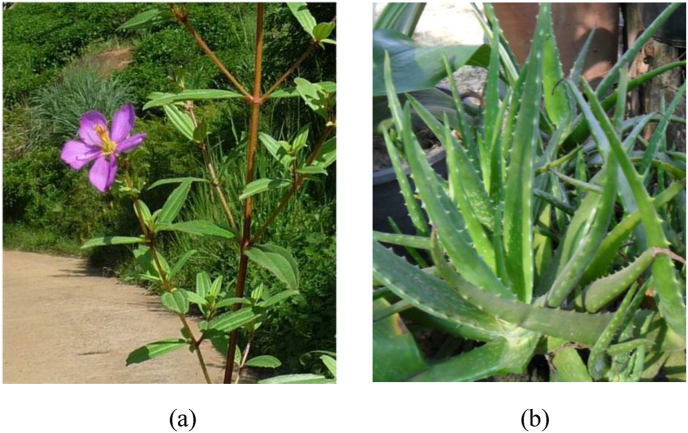
Exterior view of (a) *Osbeckia octandra*, (b) *Aloe vera*.

*Osbeckia octandra* and *Aloe vera* were authenticated by the National Herbarium of Peradeniya, Sri Lanka. The voucher specimens were deposited in the herbarium at the Research and Development Laboratory, Hettigoda Industries (Pvt) Ltd, 33/3, Sri Dharmarama Road, Ratmalana, Sri Lanka, for future reference (Fig. 1).

### 2.2 Preparation of the treatment

Liv-Pro is a food supplement in capsule form primarily formulated using the whole plant (excluding the root) of *Osbeckia octandra* (major portion) and *Aloe vera* (minor portion). The formulation utilized dried *Aloe vera* gel powder. This formulation was developed in accordance with the Ayurvedic principle of 'Dravya Samayoga Guna' which highlights the synergistic interactions between plants to enhance therapeutic efficacy. This unique composition is deeply rooted in the traditional knowledge of the Hettigoda Ayurveda family, which has a documented history of over 200 years in Ayurvedic practices. For the past 30 years, Liv-Pro formulation has been utilized in both inpatient and outpatient clinics at the Siddhalepa Ayurveda Hospital (Pvt) Ltd. It is recognized as a proprietary formulation of the Hettigoda family, reflecting the integration of traditional Ayurvedic wisdom into contemporary therapeutic applications. *Osbeckia octandra,* which is a member of the family Melastomataceae is an endemic plant species of Sri Lanka [22,23]. In the Sinhalese language, this plant is named ‘Heen Bovitiya’, and in Tamil, it is named ‘Kathtoo Mukhtohulai’ (Table 1). This plant's leaf, stem, and root are used for several liver diseases by the Sri Lankan Indigenous medicinal system. The plant is administered as herbal porridge, herbal tea, fresh juice, or sambal, made from both young and mature leaves. Additionally, its leaves, stems, and roots are used to prepare a decoction for treating liver diseases [[24], [25], [26], [27]]. *Aloe vera* is also traditionally used for liver diseases in Sri Lanka as it has a cooling and anti-inflammatory effect on the liver. The leaf gel is consumed raw or incorporated into drinks [28]. *Aloe vera* is named ‘Komarika’ in Sinhalese and ‘Angai’ in Tamil. This plant belongs to the family Asphodelaceae (Liliaceae) (Table 1).

**Table 1:** Ayurvedic properties of *Osbeckia octandra* and *Aloe vera*.

Ayurvedic Properties	*Osbeckia octandra*	*Aloe vera*
Rasa	*Amla, Madhura, Kashaya*	*Tikta, Madhura*
Guna	*Guru, Srigdha*	*Guru, Snigdha, Pichchila*
Veerya	*Sheetha*	*Sheeta*
Vipaka	*Katu*	*Katu*
Prabhava	*Lekhana*	*Bhedhana*
Action	Lekhana (scraping). This plant aids in cleansing srothas and effectively removes fat and other toxic substances from liver cells and brings the liver back to a normal state.	This plant contains anti-inflammatory, analgesic, and wound healing properties while it can relieve burning sensations and has the potency to alleviate the three doshas.

The raw materials of the plants for the manufacturing process are sourced from approved suppliers and/or company-owned plantations. Upon receipt, both herbs undergo inspection by an expert committee comprising Ayurvedic doctors, production personnel, quality control, and laboratory staff with over 20 years of expertise. This inspection ensures compliance with quality standards and the absence of contaminants, such as sand and extraneous matter. Only raw materials that meet these standards are accepted into the company stores. The stored raw materials are then released based on production requirements. These materials are washed following standard operating procedures, dehydrated, and re-inspected by the expert committee. The dehydrated herbs are subjected to size reduction through grinding and pulverization, sieved to achieve the desired particle size, and inspected again. The processed herbs are weighed and blended according to the specific formulation. The mixture undergoes a secondary drying process, after which moisture content and other critical parameters are assessed in the laboratory. The dried mixture is then steam sterilized and passed through a metal detector to ensure safety. Following this, the product is filled into containers, with inspections conducted to verify weight and microbiological parameters. Products that meet all quality requirements advance to the final packing stage, where each vegetarian capsule is carefully packaged to contain 475.0 ± 25 mg (powder + empty capsule) of the herbal formulation. The capsules then undergo a final inspection by the quality assurance department to ensure compliance with established standards. The entire manufacturing process takes place in facilities and storage units registered with the Department of Ayurveda, the regulatory authority in Sri Lanka. Additionally, Liv-Pro is also registered with the Department of Ayurveda. The company maintains compliance with several internationally recognized quality standards, including certifications from the Sri Lanka Standards Institute (SLSI), ISO 9001:2015 (Quality Management System), ISO 22000:2018 (Food Safety Management System), ISO 22716:2017 (Good Manufacturing Practices for Cosmetics), SLS 143:1999 (GMP for Food Hygiene), ISO 45001:2018 (Occupational Health and Safety), and ISO 14001:2015 (Environmental Management System).

A placebo containing roasted rice powder was used as the control in the clinical trial, which was identical in appearance and weight to the Liv-Pro capsules to ensure comparability in the trial.

### 2.3 Clinical trial design

The study was a prospective, double-blind, randomized clinical trial with two parallel treatment groups. Participants, investigators, medical professionals, technicians, and statisticians were blinded to the supplements used until after the statistical analyses were completed. The samples were given randomly to the participants. The random allocation sequence was generated by a computer program, ensuring unbiased assignment of participants as presented in.

### Ethics Committee approval

All the study-related documents were reviewed and approved by the ethics review committee of the University of Kelaniya, Sri Lanka (Number: UOK/ERC/FS/20/011) before embarking on the study. As such all procedures of the study were performed in compliance with relevant laws and guidelines of the ethics review committee. All participants provided written informed consent before recruitment to the study.

### 2.4 Study population

**Fig. 2:**
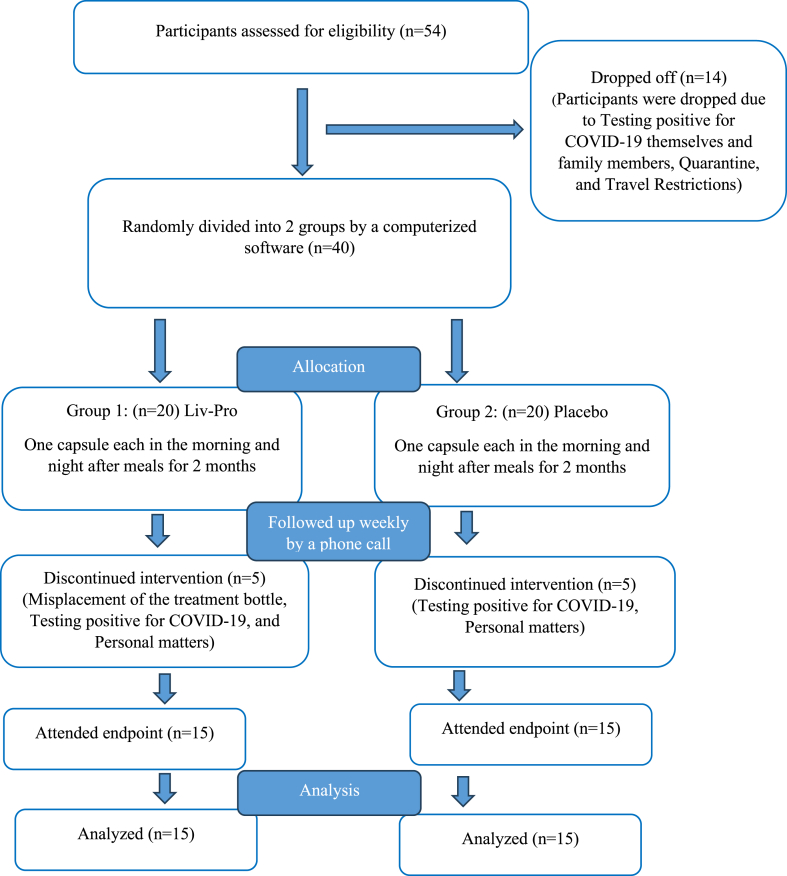
CONSORT flow Chart of the clinical trial.

Fifty-four patients were initially recruited for the study, but 24 did not complete it. Of the 24 participants, 14 participants were dropped off at the start due to Coronavirus disease of 2019 (COVID-19) restrictions. The remaining 40 patients with NAFLD received treatment at Siddhalepa Ayurveda Hospital in Mount Lavinia, Sri Lanka. However, 10 of these participants discontinued before reaching the endpoint, leaving 30 participants who completed the trial. Recruitment was conducted from 1st February, 2021, to 31st March, 2023. All participants were volunteers (Fig. 2).

#### 2.4.1 Inclusion criteria

Inclusion criteria were as follows: (1) age between 25 and 65 years, (2) either male or female, (3) previously diagnosed with NAFLD by imaging reports and with previous evidence of NAFLD according to liver profiles, (4) exhibiting lifestyle-related risk factors such as a high-fat diet and/or sedentary behavior, and/or elevated BMI, (5) not currently taking any other food supplements for NAFLD at the time of recruitment, and (6) participants from multiple locations across Sri Lanka.

#### 2.4.2 Exclusion criteria

The exclusion criteria were as follows: (1) history of significant alcohol consumption, (2) pregnancy or lactation, (3) previous history of hepatitis C liver disease, autoimmune hepatitis, viral hepatitis, liver cancer, or severe metabolic disorders, (4) participants with end-stage NAFLD such as cirrhosis, (5) individuals taking hepatotoxic medications, and (6) participants who refused to sign the informed consent form.

### 2.5 Screening method

All patients selected for the study presented with medical reports that were taken within a time frame of a few days to one week before they participated in the clinical trial. Each patient was physically evaluated by a medical practitioner prior to recruitment. Only patients previously diagnosed with NAFLD through imaging tests and supported by medical test results (including ALT and AST results) indicating evidence of NAFLD were recruited. It should be noted that, while most NAFLD cases can be diagnosed non-invasively with available medical tests, liver biopsy remains the gold standard for diagnosis [9].

### 2.6 Data collection

Data collection was carried out within the recruitment period, and all the measurements were collected during this period. All patients were personally interviewed using a questionnaire to gather details on demographic data, clinical data, lifestyle data, and any details regarding NAFLD. All selected participants had similar characteristics (Table 2).

**Table 2:** Demographic and lifestyle details of patients at baseline.

	Liv-Pro	Placebo
Number of patients	15	15
Mean age in years	46.9	42.9
Gender (Male: Female)	11:4	11:4
**Ethnicity**		
Sinhalese	13	13
Tamil	2	2
Muslim	–	–
**Occupation status**		
Engaged in an occupation	14	13
Not engaged in an occupation	1	2
**Medical history**		
Hypertension	5	1
Dyslipidemia	8	7
Diabetes mellitus	5	2
Stroke	–	–
Metabolic syndrome	1	–
Kidney disease	1	–
None of the above medical issues	1	5
**Under medication**		
Anti-cholesterol medication	4	5
Anti-diabetic medication	3	2
Anti-hypertensive medication	4	1
Family history of NAFLD	1	–
**Psychological and physical symptoms associated with NAFLD**		
Allergies	1	1
Confusion	–	–
Tiredness	–	1
Weakness	–	–
Irritability	–	–
Stress	2	–
None of the above symptoms were found	13	14
**Smoking**		
Never smoked	12	15
Occasional smoker	2	–
Heavy smoker (daily)	–	–
Stopped smoking recently	–	–
Stopped smoking many years ago	1	–
**Alcohol consumption**		
Never consumed alcohol	7	8
Occasional drinker	8	7
Heavy drinker (daily)	–	–
Stopped alcohol recently	–	–
Stopped alcohol consumption many years ago	–	–
**Diet**		
Vegetarian	1	–
Non-vegetarian	14	15
**Frequently consumed fatty food items**		
Butter	4	9
Cheese	3	6
Eggs	12	14
Milk	10	12
Meat	10	12
Fish	12	12
Chocolates	4	5
Biscuits/cookies	2	2
Cakes	4	3
Ice cream	3	2
Pastries	2	1
None of the above fatty food items are consumed frequently	0	0
**Frequently consumed fast food items**		
Fried rice	5	4
Fried noodles	5	3
Kottu roti (wheat flour mixed with oil, vegetables and/or meat and/or cheese)	4	4
Hoppers/Egg hoppers (similar to pancakes)	3	2
Chips/Fries	1	1
Burgers	2	1
Sugary drinks	1	1
Fruit juices	1	–
Instant foods	2	–
None of the above fast-food items are consumed frequently	9	10
**Conducting physical activity/exercise**		
Yes	7	6
No	8	9
**If yes frequency of physical activity/exercise**		
Once a week	4	–
Two to four times a week	1	4
More than four times a week	2	2
**Type of exercise**		
Walking	4	3
Cycling	1	–
Treadmill use	2	1
Flexibility exercises	–	2

### 2.7 Intervention

The selected participants were randomly divided into two groups where each group had 20 participants at a 1:1 ratio at the beginning of the study (Fig. 2). One group received the active treatment while the other received the placebo treatment. The sample size was calculated by a software program named G power calculator (α = 0·05; β = 0.95; and 2 comparison groups were used). Each participant received two capsules once a day, for two months. The capsule was taken during the morning and at night after meals. The study duration was limited to two months due to resource constraints and considerations of participant compliance, as extended participation time could lead to decreased retention. The patients underwent a medical assessment at baseline, at the end of the first month, and second month. During the initial visit, all participants in both groups received appropriate recommendations about dietary and physical activity, which mainly focused on the use of three to four vegetable and fruit varieties a day. Participants were also advised to conduct moderate exercises such as walking at least 20 minutes daily. The active agent, the control's safety profile, and the dose had been verified in previous studies by the investigators and by the long Ayurvedic and traditional medicine practice of the Hettigoda family. All participants were requested to bring empty bottles or unused capsules at the end of the study to achieve compliance with the treatment protocol. All participants were given a diary to maintain regarding their daily bowel habits or any side effects, which was returned at the end of the study period and if they missed a dose, they were also asked to notify about it. Each participant was followed up by a phone call at weekly intervals for awareness of any adverse reactions and to be reminded of the supplement's consumption and adherence to the trial protocol until the end of the treatment period.

### 2.8 Clinical assessment

#### 2.8.1 Primary outcomes

Medical assessments were carried out at baseline (just before the intervention), after one month and after two months. As biomedical assessments, serum liver enzyme levels such as ALT and AST, lipid profile, and serum creatinine, were carried out. The lipid profile included total cholesterol levels, low-density lipoprotein (LDL), high-density lipoprotein (HDL), and triglyceride levels. Each participant was also assessed by an abdominal ultrasound scan, which confirmed the stage of NAFLD by imaging. To obtain an unbiased quantification, all the above-mentioned medical tests were obtained from Sri Lanka Accreditation Board (SLAB) certified medical testing laboratories from various parts of the country.

#### 2.8.2 Secondary outcomes

BMI, waist circumference, and blood pressure were measured by a qualified medical professional.

### 2.9 Statistical analysis

All statistical analyses were performed using Python (Pandas, NumPy, SciPy) software. A comprehensive descriptive analysis was carried out to identify possible relations and patterns among the tested parameters. In finding the significant effect of the treatments, appropriate statistical tests were employed. A two-sided p-value (herein referred to as p) was considered significant in analyses by setting a 95 % confidence level.

## 3 Results

### 3.1 Demographic data

In the present study, 54 patients were recruited, and out of that, 14 were excluded initially due to various reasons such as testing positive for COVID-19, quarantine measures, and travel restrictions imposed by the pandemic. Thus, 40 patients with NAFLD were selected for the study, which was the minimum required sample size. Out of the 40 patients, 10 patients discontinued the intervention due to personal matters, misplacement of the treatment bottle, and testing positive for COVID-19, thus, 30 patients were analyzed. The baseline demographic details, along with clinical and lifestyle details of all 30 patients are illustrated in Table 2. All of them completed the study and their clinical data were available for the analysis. The study's outcomes were reported using the CONSORT statement criteria.

### 3.2 Clinical data- primary outcomes

Confidence intervals for various blood parameters were calculated for the two treatments. The normality of the sample data was assessed using by Shapiro-Wilk and Kolmogorov-Smirnov tests, which indicated a non-normal distribution across all parameters (p = 0.0000). Depending on the data patterns and skewness standard transformations (Log, Square, Invers, Square-root, and Box-Cox transformations) have been applied to obtain the normality. However, the Shapiro-Wilk Test has illustrated non-normality (p < 0.05). Thereby, the analysis was carried out using non-parametric statistical tests.

The standard bootstrap methods with 50 replicates were employed to calculate 95 % confidence intervals. Table 3 presents the confidence interval analysis of the means.

**Table 3:** Confidence intervals for means of the clinical data.

Clinical Parameter	Treatment Type	Treatment Duration	(Mean, SD)	Confidence Interval at 95 % for Mean (Bootstrap)
Total cholesterol	Placebo	Initial	(210.2, 39.0)	(191.8, 230.1)
		After 1 month	(217.1, 28.6)	(202.9, 230.7)
		After 2 months	(223.8, 34.4)	(206.7, 240.4)
	Liv-Pro	Initial	(218.5, 33.0)	(203.2, 235.3)
		After 1 month	(188.2, 26.2)	(175.0, 201.6)
		After 2 months	(204.0, 41.2)	(184.4, 223.4)
ALT/SGPT	Placebo	Initial	(50.5, 33.0)	(35.9, 68.1)
		After 1 month	(54.3, 35.4)	(38.9, 73.8)
		After 2 months	(51.0, 41.2)	(34.0, 73.9)
	Liv-Pro	Initial	(89.0, 50.7)	(66.1, 115.3)
		After 1 month	(70.9, 37.0)	(53.1, 90.7)
		After 2 months	(64.3, 39.1)	(46.8, 84.9)
AST/SGOT	Placebo	Initial	(30.9, 9.6)	(26.3, 35.8)
		After 1 month	(34.2, 13.9)	(28.1, 41.6)
		After 2 months	(32.8, 11.7)	(27.3, 39.0)
	Liv-Pro	Initial	(52.5, 24.9)	(41.1, 65.3)
		After 1 month	(48.4, 31.4)	(34.3, 64.9)
		After 2 months	(44.6, 33.7)	(31.0, 63.4)
Triglycerides	Placebo	Initial	(147.0, 48.9)	(124.2, 173.2)
		After 1 month	(136.1, 36.6)	(119.0, 154.6)
		After 2 months	(138.3, 40.9)	(118.5, 158.2)
	Liv-Pro	Initial	(182.2, 85.2)	(143.1, 227.3)
		After 1 month	(147.0, 68.4)	(113.4, 183.0)
		After 2 months	(150.7, 66.3)	(118.8, 183.6)
LDL	Placebo	Initial	(134.2, 37.2)	(115.8, 152.6)
		After 1 month	(145.7, 28.3)	(131.2, 159.2)
		After 2 months	(144.1, 46.7)	(123.4, 168.9)
	Liv-Pro	Initial	(128.0, 34.7)	(111.6, 145.2)
		After 1 month	(112.6, 26.1)	(99.2, 125.3)
		After 2 months	(117.5, 33.0)	(101.9, 134.3)

**Fig. 3:**
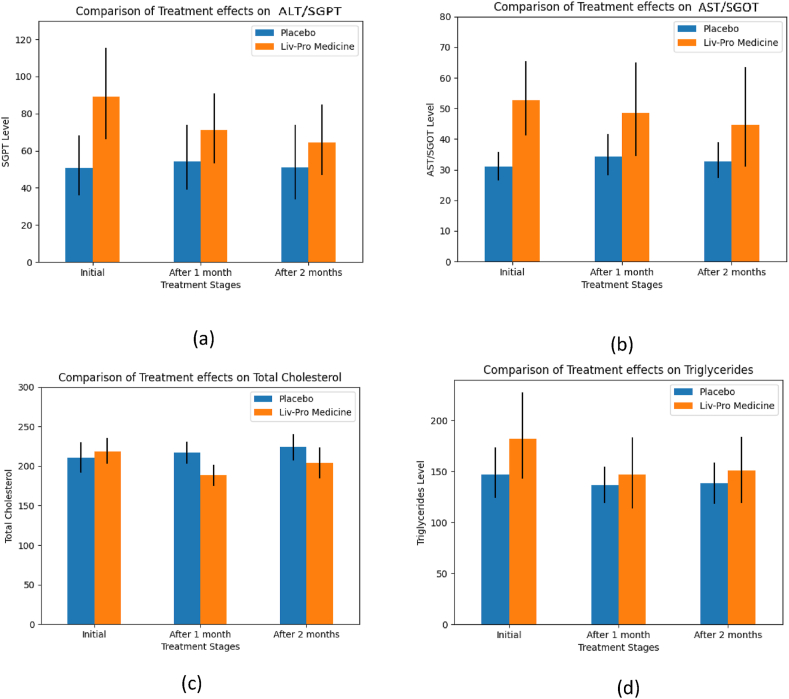
Comparison of the respective means of the clinical parameters under the Liv-Pro treatment (a) ALT/SGPT, (b) AST/SGOT, (c) Total Cholesterol, (d) Triglycerides. The black line shows the confidence interval limits.

**Fig. 4:**
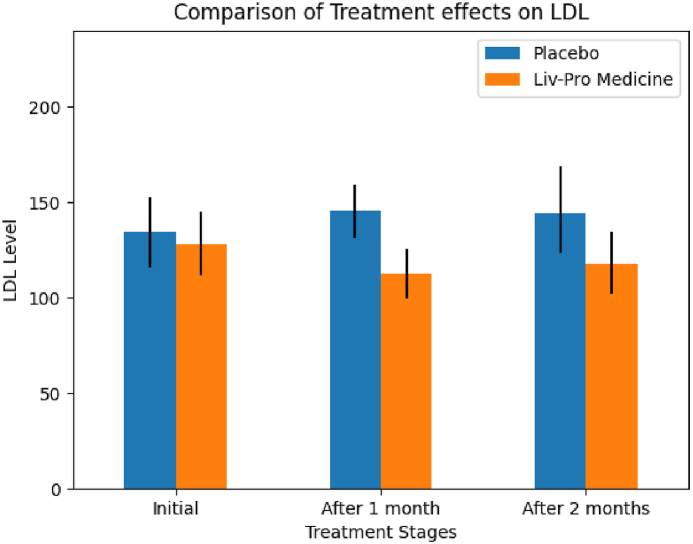
Comparison of the mean of LDL under the Liv-Pro treatment. The black line shows the confidence interval limits.

Most clinical parameters responded within the first month of treatment. Notably, ALT and AST exhibited high sample variance, whereas other parameters showed moderate variance in comparison. The high variance in ALT and AST can be attributed to differences in patient characteristics, including age, severity of illness, and other factors. However, this variability enables the assessment of treatment effects across a broader range of patients, enhancing the validity of the findings. Furthermore, the decrease in total cholesterol levels indicates a positive treatment response Fig. 3. Similarly, Table 4, Fig. 4illustrates a reduction in LDL levels, suggesting that Liv-Pro contributed to lowering 'bad' cholesterol (LDL).

Table 4, presents a summary of the clinical data analysis for ALT, AST, total cholesterol, LDL, triglyceride levels, serum creatinine, and the results of the abdominal ultrasound scan. A comparison of treatment effects between Liv-Pro and placebo was conducted using p-values obtained from significance tests. Given the heterogeneity of the subject groups and other factors, the data did not meet the normality assumption. Therefore, nonparametric techniques were applied for analysis. Biochemical values measured at baseline (before the initiation of treatments) were compared to those measured after one-month and two-month periods. These time intervals, representing baseline values, one-month values, and two-month values, serve as treatment stages for statistical comparative analysis. The Friedman test (nonparametric ANOVA for related samples) was utilized to assess the presence of any progressive treatment effects over time, and the Wilcoxon Signed Rank test was employed as a post-hoc test to compare the pairwise effects and the differences. This analysis was conducted for both the Liv-Pro and placebo patients. Despite the insignificance of all patients on biochemical parameters in the placebo group, p-values were provided to illustrate the significance given by tests. All the tests were conducted with 95 % confidence (significance ***α*** = 0.05).

**Table 4:** Clinical data of all patients compared to baseline measurements and after one month and two months of treatment.

Test	Liv-Pro				Placebo			
	Treatment Effect Significance (Friedman)	After one month (Wilcoxon Sign Rank Test)	After two months (Wilcoxon Sign Rank Test)	First month to second month (Wilcoxon Sign Rank Test)	Treatment Effect Significance (Friedman)	After one month (Wilcoxon Sign Rank Test)	After two months (Wilcoxon Sign Rank Test)	First month to second month (Wilcoxon Sign Rank Test)
ALT/SGPT	0.049	0.049	0.048	0.573	0.342	0.323	0.428	0.339
AST/SGOT	0.034	0.047	0.046	0.267	0.581	0.512	0.676	0.722
Total cholesterol	0.022	0.003	0.048	0.101	0.422	0.543	0.411	0.533
Triglycerides	0.017	0.061	0.041	0.061	0.701	0.791	0.693	0.733
LDL	0.049	0.049	0.047	0.161	0.334	0.431	0.211	0.899
Serum creatinine	0.516	0.426	0.245	0.865	0.597	0.776	0.675	0.421

ALT and AST are liver function tests. ALT is generated primarily by the liver whereas AST is generated by various organs such as the liver, heart, kidneys, and the brain. Hepatocytes of the liver produce ALT and AST, and elevated ALT and AST in the blood are laboratory markers indicating hepatocellular damage and inflammation [29]. These enzyme levels are frequently evaluated in the context of NAFLD to measure the severity of the condition. As shown in Table 4, Liv-Pro was able to reduce the ALT levels. Post-hoc tests indicated that ALT levels were significantly lower after one month (p = 0.049) and after two months (p = 0.048) compared to baseline measurements. However, no significant difference was observed between the effects from one month to two months (p = 0.573). Similar to ALT, Liv-Pro demonstrated a positive impact on controlling AST levels as well. Interestingly, the significance of the Liv-Pro effect on AST was higher than that of ALT (lower p = 0.034 < 0.049, in treatment effect). Furthermore, comparing the p-values Liv-Pro illustrated a consistent decline in AST over time. As such AST after one month (p = 0.047) and two months (p = 0.046) were significantly lower in the Liv-Pro group.

NAFLD is frequently characterized by dyslipidemia. Most NAFLD patients show abnormalities in their lipid profile and many studies have shown that cardiovascular events are the leading cause of mortality in NAFLD patients [30]. Interestingly, Liv-Pro demonstrated a significant impact on reducing total cholesterol levels compared to other biochemical parameters considered in this study (p = 0.022). At the end of the first month, a remarkably significant outcome in total cholesterol levels (p = 0.003) was observed, and there was a continued significant effect in the second month as well (p = 0.048). Triglyceride is another lipid parameter, that has been influenced by Liv-Pro, resulting in a decline in its levels. Similar to total cholesterol, triglyceride levels decreased significantly after two months of treatment rather than one month of treatment (p = 0.041).

**Fig. 5:**
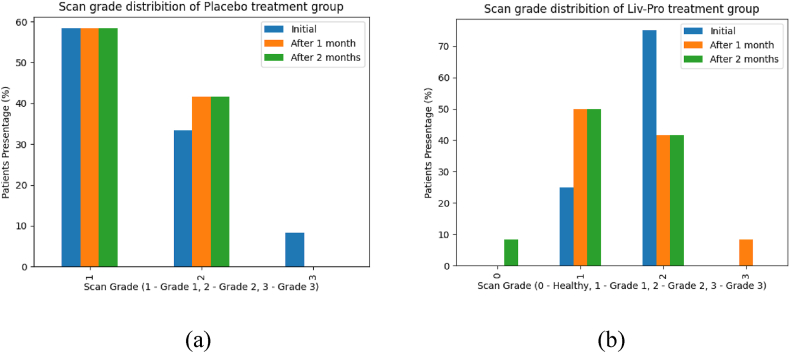
Comparison of the NAFLD (fatty liver) grades. (NAFLD grades of patients treated with placebo (a), and NAFLD grades of patients treated with Liv-Pro (b)).

Regarding LDL, the effect of Liv-Pro remained consistently low throughout the two-month treatment period, where the first-month reduction p = 0.049, and the second reduction p = 0.047, was significant compared to baseline measurements. When considering HDL, it remained at the accepted levels (40–59 mg/dL) in the Liv-Pro group (after one month lower limits p = 0.017 and upper limit p = 0.000; after two months, lower limit p = 0.002 and upper limit p = 0.000) [31]. As such based on sample observations and analysis with 95 % confidence, the study suggests that Liv-Pro can be effectively utilized for controlling total cholesterol, triglyceride, and LDL levels. Serum creatinine is a waste product secreted by the kidneys. Serum creatinine levels that are elevated can suggest compromised renal function. Some herbal treatments may pose a risk of kidney damage. Hence, monitoring serum creatinine levels is a way to assess the impact of the given medication on kidney function and identify potential kidney-related side effects [32]. Therefore, the serum creatinine levels of all patients who were given the treatment were tested. All patients exhibited optimal serum creatinine levels at the onset of the study. Upon comparing serum creatinine levels throughout treatment, no evidence was found to suggest any significant changes during the two months with a 95 % confidence level. Abdominal ultrasound scans can detect and quantify the presence of fat in the liver. This is critical in diagnosing fatty liver disease, characterized by aberrant fat accumulation in liver cells. Further, imaging enables the grading of fatty liver disease based on the amount of fat accumulation [9]. Therefore, the grade of the disease was determined by an abdominal ultrasound scan Fig. 5. Fig. 2, illustrates the percentage distribution of NAFLD grades for the treatment (Liv-Pro) and control (placebo) groups.

At the initial stage, 75 % of patients treated with Liv-Pro presented with grade 2 NAFLD, while 25 % had grade 1. Notably, there were no healthy individuals (with respect to NAFLD) or grade 1 patients in this cohort. After one month of treatment, the proportion of grade 2 patients decreased to 42 % due to improvements in their condition, while 10 % of patients progressed to grade 3. However, after two months of continuous treatment, the outcomes improved significantly: 10 % of patients were classified as healthy (NAFLD-free), and all grade 3 patients showed improvement to grade 2. In contrast, the placebo group exhibited no overall improvement, except for a 10 % reduction in grade 3 cases, with these patients improving to grade 2. This highlights the efficacy of Liv-Pro in enhancing liver health and mitigating NAFLD progression. To statistically validate these findings, Kendall's test was used to assess the proportional improvement between the Liv-Pro and placebo groups. The test yielded a significant result (p = 0.020), indicating that Liv-Pro contributed to a reduction in fatty liver grade over two months compared to baseline. In contrast, none of the placebo group parameters showed significant improvement in primary outcomes (p > 0.05).

### 3.3 Clinical data- secondary outcomes

No significant evidence was found at the conclusion of the trial in blood pressure; both upper and lower limits (p = 0.766, 0.653, respectively). Further, mean BMI was reported as 27.4 in the Liv-Pro group while it was reported as 26.5 in the placebo group. The results were not significant at the end of the trial; BMI (p = 0.788). Furthermore, the mean waist circumference was 98.3 cm in the Liv-Pro group while it was reported as 96.9 cm in the placebo group. The results were not significant at the end of the trial (p = 0.673). None of the placebo parameters were significant in the secondary outcomes (p > 0.05).

## 4 Discussion

At present, the only effective treatment for NAFLD is a lifestyle change. Nevertheless, it has been demonstrated that the aforementioned NAFLD management technique is largely ineffective over an extended length of time [12]. Liv-Pro capsules are a liver-specific herbal formulation meant to treat diseases of the liver, particularly NAFLD, which can act against the progression of NAFLD. To our knowledge, this is the first-ever clinical trial conducted with the combination of the medicinal plants *Osbeckia octandra* and *Aloe vera* against NAFLD. The liver is an incredibly vital organ in the human body, performing a multitude of essential functions. It is responsible for metabolism, detoxification of various metabolites, synthesizing proteins, and storing nutrients to support overall health [33]. Liv-Pro could also be used as a preventative measure or as a supplement alongside hepatotoxic medications to protect the liver. Before initiating the clinical trial, extensive laboratory research was conducted to assess the efficacy of the Liv-Pro formulation at the Hettigoda Industries Research and Development laboratory to obtain scientifically validated results. Through this research, various formulations were evaluated of the selected plants. The combination of *Osbeckia octandra* (major portion) and *Aloe vera* (minor portion) yielded the most promising scientific results in the laboratory. The combined application of both plants exhibited the most pronounced levels of bioactivity, particularly in terms of anti-oxidant and anti-inflammatory properties, as determined through *in vitro* bioassays. Furthermore, the analysis revealed a notable concentration of phenolic compounds within the formula, with gallic acid identified as one of the major constituents, a chemical compound found as a secondary metabolite in *Osbeckia octandra*. Gallic acid is found to have hepatoprotective effects due to its high antioxidant and anti-inflammatory properties. Research shows that gallic acid can decrease plasma AST and ALT activities by preventing the leakage of intracellular enzymes by stabilizing the cell membrane and improving the antioxidant status in liver cells. Due to being an antioxidant, gallic acid has been hypothesized to exert its effects on NAFLD by acting as a scavenger for free radicals generated during lipid peroxidation, thereby disrupting the propagation of oxidative reactions [[34], [35], [36], [37], [38], [39]]. Nevertheless, it is plausible that additional bioactive compounds may contribute to the hepatoprotective effects.

In the current study, the results showed that Liv-Pro can reduce the liver enzymes ALT and AST. ALT is a key marker of liver function, and the observed decrease indicates an improvement in hepatic health. The attenuation of liver enzyme activity suggests that Liv-Pro may play a role in mitigating liver inflammation and damage via its anti-oxidant and anti-inflammatory mechanisms. Further, abnormal lipid levels have been shown to alter liver metabolism, potentially causing damage to hepatic tissue [40]. Research indicates that patients with NAFLD often exhibit hypercholesterolemia, characterized by elevated levels of total cholesterol, low-density lipoprotein (LDL), and triglycerides, along with decreased high-density lipoprotein (HDL). This dyslipidemic profile contributes to the increased cardiovascular risk observed in NAFLD patients [41]. Notably, there is a link between hypertension, aberrant lipid profiles, and NAFLD, with NAFLD patients experiencing a significantly higher mortality rate than the general population due to cardiovascular illness [11]. These conditions frequently coexist, driving each other's development and progression. In the present study, Liv-Pro was found to effectively lower lipid profiles, thereby mitigating the risk of cardiovascular disease [42]. Previous studies have shown that NAFLD increases hypertension, following cardiovascular diseases [43]. The administration of Liv-Pro did not result in any significant alteration of blood pressure levels among the patients, with no observed increase or decrease in these levels. There was also no change in BMI and waist circumference during the two months. Imaging studies are a non-invasive method for evaluating NAFLD [44]. Abdominal ultrasound scans revealed a noticeable reduction in hepatic fat content among those who used Liv-Pro after two months of treatment. In fact, preclinical research has shown that aqueous extracts of *Osbeckia octandra* ameliorate liver fibrosis in cirrhotic rats through the inhibition of pro-inflammatory and pro-fibrotic cytokine secretion, as well as angiogenesis, highlighting its potential therapeutic effects on liver pathology [45]. This is particularly remarkable for individuals with NAFLD and suggests a potential benefit in managing this condition. Serum creatinine levels, a crucial indicator of kidney function, remained within normal ranges throughout the trial, indicating that Liv-Pro supplementation did not adversely affect renal function [46]. This is an essential aspect of safety and suggests that the supplement is well-tolerated in the study population.

In this study, participants in the Liv-Pro group showed promising outcomes, including improved liver enzymes, reduced total cholesterol, triglyceride, and LDL levels, as well as decreased liver ultrasound grades compared to the placebo group. These results suggest Liv-Pro's potential as a beneficial and effective dietary supplement for individuals with fatty liver. The supplement was well-tolerated, with no reported adverse effects or gastrointestinal issues among participants during the trial period. However, the study had limitations such as short evaluation time points, a limited number of study participants, and challenges due to the COVID-19 pandemic, leading to difficulties in participant recruitment and completion. Future research may benefit from larger trials with longer intervention periods to further explore Liv-Pro's efficacy. However, it should be noted that the Liv-Pro formulation, rooted in the traditional expertise of the Hettigoda family with a 200-year legacy in Ayurvedic medicine, has been effectively utilized at the Siddhalepa Ayurveda Hospital for over three decades in both inpatient and outpatient care. Its longstanding and consistent use in clinical settings provides sufficient evidence of its safety, which may reduce the necessity for prolonged follow-up evaluations.

## 5 Conclusions

Liv-Pro, a food supplement designed to support liver health, has demonstrated promising results in a recent double-blind randomized clinical trial with two parallel treatment groups involving patients with NAFLD. The trial aimed to evaluate the efficacy and safety of Liv-Pro in improving liver function and reducing key markers associated with liver damage. Therefore, the present study revealed that treatment with Liv-Pro led to a notable enhancement in liver function parameters, specifically AST and ALT, accompanied by reductions in total cholesterol, triglyceride, and LDL levels, at 95 % confidence. Additionally, there was an improvement in liver ultrasound scan grades after two months of treatment. Further, there were no clinically significant adverse effects reported during the clinical study. The findings from this clinical trial suggest that Liv-Pro could serve as a beneficial dietary adjunct for individuals focusing on liver health. Its demonstrated capacity to lower liver enzymes, decrease hepatic fat content, and enhance lipid profiles highlights its potential effectiveness in managing NAFLD. Liv-Pro presents itself as a promising measure for liver wellness and enhances good health and well-being when utilized as a food supplement and. it is important to note that continual use of Liv-Pro along with a healthy lifestyle may yield better results in maintaining liver health over time.

## Ethics approval and consent to participate

The study adhered to the principles outlined in the Declaration of Helsinki for all procedural protocols. Before recruitment, all participants furnished written informed consent. Approval for the study protocol was obtained from the ethical committee of the University of Kelaniya, Sri Lanka (Number: UOK/ERC/FS/20/011).

## Author contribution

The study design was formulated by AIK, PAP, and LH. AIK, AC, PAP, and LH were involved in the execution of the trial. Statistical analysis and interpretation of results were conducted by UPL and PAP. The initial manuscript was prepared by AIK. Subsequent revisions and critical feedback on the manuscript were provided by UPL, PAP, and LH, leading to successive drafts by AIK. The final version of the manuscript was approved by all authors.

## Declaration of generative AI in scientific writing

The authors declare that no AI tools were used for the writing of the manuscript.

## Funding

Hettigoda Industries (Pvt) Ltd, Ratmalana, Sri Lanka, funded the entire clinical trial.

## Declaration of competing interest

All authors declare that there are no competing interests.
